# Phase Stability under Thermal Drifts in Photodiode-Conditioning Transimpedance Amplifiers for Distance Metrology

**DOI:** 10.3390/s21103455

**Published:** 2021-05-15

**Authors:** Francisco Javier Meca Meca, Ernesto Martín-Gorostiza, Miguel Ángel García-Garrido, David Salido-Monzú

**Affiliations:** 1 Electronics Department, Polytechnic School, University of Alcalá, 28871 Madrid, Spain; francisco.meca@uah.es (F.J.M.M.); miguelangel.garcia@uah.es (M.Á.G.-G.); 2 Institute of Geodesy and Photogrammetry, ETH Zurich, 8093 Zurich, Switzerland; david.salido@geod.baug.ethz.ch

**Keywords:** transimpedance amplifiers, delay time, phase stability, thermal drifts, signal to noise ratio, distance measurement

## Abstract

Transimpedance amplifiers (TIA) are widely used for front-end signal conditioning in many optical distance measuring applications in which high accuracy is often required. Small effects due to the real characteristics of the components and the parasitic elements in the circuit board may cause the error to rise to unacceptable levels. In this work we study these effects on the TIA delay time error and deduce analytic expressions, taking into account the trade-off between the uncertainties caused by the delay time instability and by the signal-to-noise ratio. A specific continuous-wave phase-shift case study is shown to illustrate the analysis, and further compared with real measurements. General strategies and conclusions, useful for designers of this kind of system, are extracted too. The study and results show that the delay time thermal stability is a key determinant factor in the measured distance accuracy and, without an adequate design, moderate temperature variations of the TIA can cause extremely high measurement errors.

## 1. Introduction

Distance measurement by optical methods enables a large number of applications such as commercial optical rangefinders and LADAR systems [1,2,3,4,5], large-scale dimensional metrology [6], mobile robot positioning [7,8] and Time-of-Flight Cameras [9,10,11]. In a typical range-measurement setup, distances are derived from the round-trip propagation delay of an optical signal transmitted from a source and back reflected by a passive target. For medium and large distances (from meters to some km), there are two main methods used for this purpose [5,12]: Time of Flight (TOF) and phase-shift or continuous-wave measurements. Depending on the approach, the distance *d* between source and target is obtained either from a TOF (*T_F_*) or from a phase (*ϕ*) measurement, making use of the well-known telemetry relations, 2*d* = *T_F_* · c or 2*d* = *ϕ* · c / (f · 2π), respectively, where c is the average speed of light, *ϕ* is the phase shift corresponding to the distance *d* along the propagation path (typically approximated from standard or measured meteorological conditions at the instruments’ location), and time *T_f_* is the TOF corresponding to the path travelled, and *f* is the optical intensity modulating frequency.

**Figure 1:**
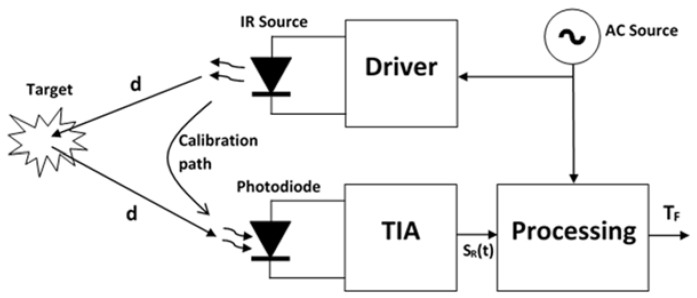
Diagram of optical distance measurement system where emitter and receiver share the same location. Inline recalibration possible through internal calibration path.

In Figure 1, a generic distance measuring system is depicted, showing a typical source such as an infrared (IR) emitter and also a photodiode to receive the reflected signal from the target, followed by a trans-impedance amplifier (TIA) plus a processing unit to obtain *T_F_* or *ϕ* and deduce the value of *d*. This structure is valid for any of the two methods mentioned in the previous paragraph.

The distance information is contained in the delay (or phase shift) between the emitted and received signals. This phase shift is affected by errors due to both random (noise) and systematic contributions. Among the systematic errors, we can distinguish between constant deviations (due to tolerances, circuit path lengths, etc.) and error drifts, typically dominated by thermal variations. The impact and mitigation of the latter is the main focus of this work. 

The error caused by noise depends on the SNR and can be reduced so that high precisions are achievable with a careful design [8]. This aspect has been thoroughly addressed in the literature on this topic [13,14]. Regarding systematic error contributions, the phase shift measured includes the delay time caused by the emitter and receiver electronic circuits. This delay depends on the electronic circuit parameters, namely bandwidth, which in turn depends on the tolerances of the elements in the circuit and must be known in order to remove it from the measurement. This is typically done with a system calibration procedure in which a fraction of the emitted signal is deviated to the photodetector through an optical calibration path. The delay time obtained in this way is the sum of the electronic circuit delay explained before and the optical calibration path, which is a known value. High accuracies are achievable with this procedure [12]. Once the constant deviations due to electronic delays and optical path are removed from the measurement, electronic drifts (mostly thermal) remain as a variable error source. Consequently, periodic recalibration is needed to eliminate this error and preserve the accuracy needed. Although in many applications inline recalibration is possible, keeping the error within the required accuracy margin, it is often inconvenient to do it frequently since the system is not operative during the calibration measurements.

**Figure 2:**
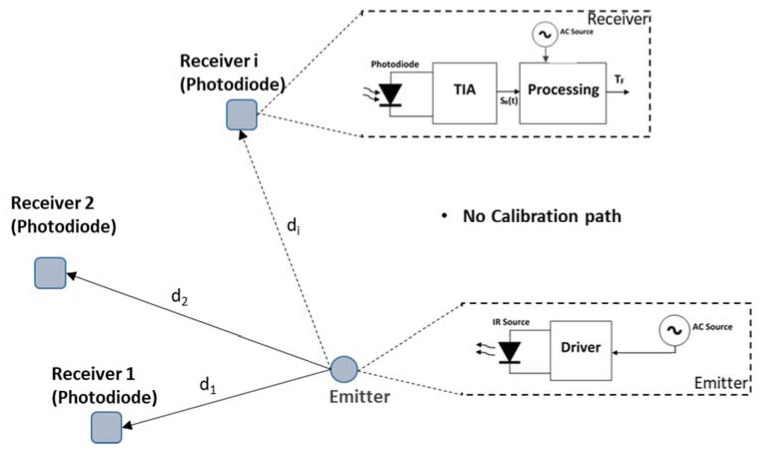
Optical distance measurement system with remote receivers where phase drifts may be critical.

Moreover, in some applications, this recalibration process is extremely complicated. This is especially the case when the photodetector and the emitter (source) do not share the same location, as represented in Figure 2. A system like this entails high practical complexity, and so, once calibrated, it is desirable that the accuracy keeps within the required margin under the expected temperature variations in the working environment of the system. An important application of this kind is, for example, a positioning system where several receivers are placed at different locations [8,15]. 

The existing scientific literature about optical distance measurement typically focuses on problems related to SNR improvement and walk error reduction [16,17]. When working with systems like the one in Figure 2, where recalibration is not desirable or even not possible, the error caused by these factors can be far below those ones due to thermal drifts. In such situations, delay time stability becomes a major concern. As for this issue, in such a deployment, the critical point is the electronic unit in charge of processing the signal delivered by the photodetector. This device provides a current proportional to the received radiated power, which is customarily converted into a (proportional) voltage signal by means of a trans-impedance amplifier (TIA). In these cases, the emitter does not represent a problem, as the signal is received at all photodetectors. Therefore, any error arising at the emitter is common to all receivers, being easily removable by, for instance, making differential measurements. 

The TIA becomes a key element in the quality of the measurement. Hence, a deep analysis of the parameters of the TIA that contribute to the uncertainty in the measurement is required. For distance measurement, these parameters include equivalent noise spectral density with reference to the input and stability of the photodiode conditioning circuit delay time. The former has been thoroughly studied [13,14,18,19,20], whereas there is lack of specific works about the second one, which, as explained, is a key aspect in systems like in Figure 2.

More specifically, the analysis must take into account the two following circuit parameters that directly introduce uncertainty in the measurement: the stability of the circuit transfer function, and SNR. Regarding the first one, phase variations in the transfer function, mainly due to thermal effects, directly translate into delay time changes, hence into deviations of the measured distance. Thus, the TIA must meet certain specifications on phase stability within the temperature variation range. Regarding SNR, it translates into random phase—therefore distance—variations, thus determining the measurement precision. The improvement of SNR levels through proper design and filtering will improve precision and/or dynamic range (maximum measuring distance). Although systematic drift and random variations are error sources of different nature, in this work it will be shown that improving the drift error worsens the SNR. This trade-off must be addressed in the TIA design process.

The aforementioned parameters (transfer function and SNR) depend on the components of the TIA circuit, i.e., resistors and capacitors in the equivalent circuit of the real TIA model (including the photodetector and operational amplifier models) as well as the parasitic capacities of the printed circuit board. Thus, the analysis of the transfer function phase variations and SNR explained in previous paragraph must be extended down to these low-level circuit parameters and their thermal characteristics.

In the past, we succeeded in developing an IR distance measuring system for indoor positioning with precision in the cm level [7,8,21]. That sensor was designed under the aforementioned SNR considerations, but not the receiver electronics thermal drifts. This work focuses on the analysis and characterization of all these contributions to the thermal instability of the TIA delay time. It also proposes some guidelines to reduce them and quantifies the impact on the system SNR. The main contribution of this work lies in addressing the circuit thermal drifts as the main cause of severe measuring errors, instead of the typical approach focused only on SNR. Here, SNR is included in a comprehensive trade-off (thermal drifts versus SNR) that must be addressed at the design level. The TIA structure proposed is a classical configuration with an operational amplifier and a RC feedback network searching for the limits of phase stability. Other topologies based on capacitive feedback are interesting when high gains are needed, avoiding saturation due to the dark current, and also reaching good noise levels [22]. However, in our case, the system is proposed for indoor environments and dark current does not represent a major problem. Let us also remark that the focus of this work is not to propose a distance measuring method but to analyze the TIA structure in detail. Distance measuring is a natural, straightforward application of this circuit, but any other system which measures phase shift using this circuit topology can benefit from the study developed here.

In Section 2, the aforementioned analysis is carried out: first, in Section 2.1 we address phase stability with an ideal operational amplifier (OA) in the design, followed by the analysis of phase stability against printed circuit parasitic capacities in Section 2.2. In Section 2.3 we derive the relation between circuit parameters and SNR. Finally, phase stability considering a real OA is tackled in Section 2.4. 

As the study carried out in this section goes deep into very low-level details, we provide in Section 3 a comprehensive summary of the most important concepts addressed in this analysis, which also recalls the main design guidelines derived from the analytical study. The reader can independently read this section and/or the details provided in the previous one as desired. In Section 4 we illustrate the results of the analysis evaluating a case study and showing real measurements to validate it. Conclusions are included at the end of the paper in Section 5.

Finally, note that we will refer hereafter to phase shift or delay time interchangeably in order to avoid excessive repetition of either or both terms. Both concepts are directly related once the modulating frequency is fixed. Also note that delay time refers to the time increment, equivalent to the phase shift, in the receiver electronics, but not to the emitter-receiver TOF.

## 2. Analysis of the TIA Phase Stability and SNR

**Figure 3:**
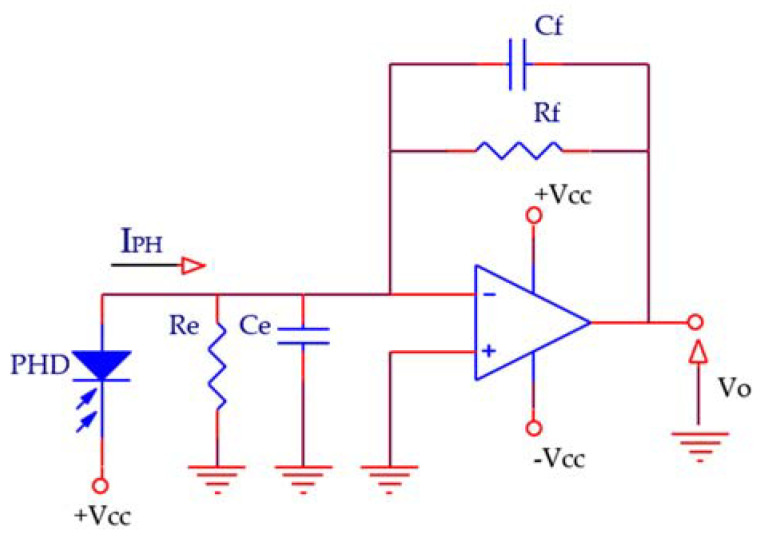
TIA model and equivalent input impedances (Re and Ce).

Figure 3 shows the schematic of a TIA based on an operational amplifier (OA), where the feedback network Rf-Cf, together with the OA and photodiode characteristics, defines the noise, bandwidth (BW) and delay time parameters. Re represents the equivalent input resistance of the OA (R_CM_//R_DM,_ being R_CM_ and R_DM_ the OA common mode and differential mode input resistances respectively) combined with the shunt resistance of the photodiode (R_PH_), i.e., Re = R_CM_//R_DM_//R_PH_. Ce includes the total equivalent input capacitance of the OA (C_CM_ + C_DM_, being C_CM_ and C_DM_ are the OA common mode and differential mode input capacitances) plus that of the photodiode (C_PH_), i.e., Ce = C_CM_ + C_DM_ + C_PH_.

### 2.1. Phase Stability Considering Ideal OA Parameters

Assuming that the OA in Figure 3 is ideal, the transfer function of the circuit follows Equation (1), where *I_PH_* is the photodiode current, *f* is the IR-intensity modulating frequency and *f_C_* is the -3dB cut-off frequency of the TIA.

(1) $$TI\left(jf\right)=\frac{Vo}{I_{PH}}\left(jf\right)=-\frac{Rf}{1+j2\pi f\cdot Rf\cdot Cf}=-\frac{Rf}{1+\frac{jf}{fc}}$$

The phase introduced by this circuit is:

(2) $$\alpha \left(f,f_{C}\right)=∠TI\left(jf\right)=\pi -arctan\left(\frac{f}{f_{C}}\right)$$

where ∠∙ denotes the argument (angle) of a complex number. Variations in the values of the feedback network composed of *Rf*//*Cf* introduce changes on the phase of the signal. We define here the stability in terms of the increment caused by the parameters deviations, thus we will refer hereafter to stability or deviation (of phase, or delay time or distance) without distinction. In this way, the phase stability Δα is:

(3) Δα(f,fC)≅dα(f,fC)dfCΔfC=ffC1+(ffC)2ΔfCfC≅−ffC1+(ffC)2(ΔRfRf+ΔCfCf)

and the measured distance stability is, thus, as follows:

(4) Δd(f,fC)=c2πfΔα(f,fC)=−c2πfffC1+(ffC)2(ΔRfRf+ΔCfCf)=−c2πfC11+(ffC)2(ΔRfRf+ΔCfCf)

**Figure 4:**
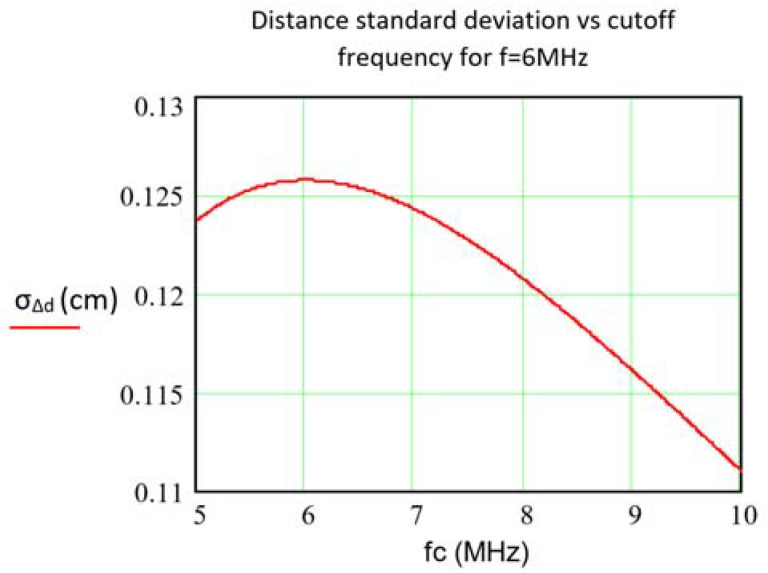
Standard deviation of distance error as a function of TIA cut-off frequency with *f* = 6 MHz, *σ_TC_(Rf)* = 10 ppm/°C, *σ_TC_(Cf)* = 30 ppm/°C and Δ*T* = +10 °C. Maximum value: σ_Δ_*_d_*(*f* = 6 MHz, fc = 6 MHz) = 0.126 cm.

Changes in these components with respect to their calibrated values are primarily introduced by thermal drifts. According to Equation (4), given certain *f_C_* the stability in the distance measurement improves with increasing the emission frequency *f*. This is shown in Figure 4, computed for the specific parameter values that will be used in the experiments described later. According to Figure 4, assuming *f_C_* > *f*, higher *f_C_* reduces the impact of relative changes on *Rf* and *Cf*. This is independent of the specific parameter values used to compute Equation (4) and enables sub-millimeter stabilities to be obtained by making *f_C_* sufficiently large.

### 2.2. Impact of PCB Parasitic Capacitance

As demonstrated above, increasing f_C_ reduces the phase sensitivity of the system to relative variations in *Cf* and *Rf*, which is achieved by reducing the product *Rf* × *Cf*. By reducing *Cf*, the parasitic capacitance (*C_FP_*) introduced by the PCB into the feedback of the OA, which can be modeled in parallel to *Cf*, will be proportionally more significant. Its variations can therefore introduce larger distance errors than due to the temperature coefficient (TC) of the capacitance *Cf*.

The thermal stability of *C_FP_* is defined by the thermal stability of the dielectric constant and the thermal expansion coefficients of the material used to manufacture the PCB. For example, for a standard FR-4 PCB, typically used for high frequency circuits, the TC of the dielectric constant is in the range of 200 to 400 ppm/°C [23]. Based on this contribution alone, *C_FP_* values only ten times smaller than *Cf* introduce as much thermal instability in the distance measurement as a high-stability *Cf* capacitor. *C_FP_* values of a few tenths of a pF are expectable on designs where the minimization of parasitic capacitances is not specifically addressed, while carefully designed PCBs are unlikely to show *C_FP_* values significantly below 0.1 pF. Given that common TIA designs for photodiodes use *Cf* values around or below 1 pF, analyzing the influence of *C_FP_* on the phase stability becomes relevant.

The distance error as a function of the absolute variation of *Cf* follows Equation (5), which can be used to calculate the maximum value of *Rf* as a function of the targeted distance error, the expected variation of the parasitic capacitance associated with *Cf* (Δ*C_FP_*), and the relationship between *f* and *f_C_*.

(5) Δd(f,fC)=c2πfΔα(f,fC)=−c2πfC11+(ffC)2ΔCfCf=cRf1+(ffC)2ΔCFP Rf≤1+(ffC)2c·ΔCFPΔd(f,fC)|Max

As follows from Equation (5), it is always possible to limit the effect of Δ*C_FP_* by reducing the value of *Rf*, i.e., by increasing the cut-off frequency *f_C_* of the system. The admissible value of *Rf* is maximum at *f* = *f_C_* if keeping the information within the amplifier BW is imposed. To maximize the SNR, which is achieved by increasing *Rf*, and to comply with the error specifications due to *C_FP_* variations, the system should therefore be designed so that *f* = *f_C_*. 

The design condition *f* = *f_C_*, which as shown in Figure 4 provides the worst design option in terms of error due to relative variations of *Cf* and *Rf*, provides the best compromise between errors due to due to *C_FP_* variations and system SNR as will be addressed further. Moreover, as can be deduced from Equation (5), it may even be of interest that *f* > *f_C_* as long as the SNR of the TIA does not degrade unacceptably above the cut-off frequency.

The parasitic capacitance *C_eP_* of the PCB between the inverting and non-inverting inputs of the OA is in parallel with the equivalent capacitance *Ce* in Figure 3. As will be seen further on in Section 2.4 and in Equation (13), the impact of the variations of this parasitic capacitance can be deduced from the analysis of the effect of *Ce* variations. With a moderately good design of the PCB, this capacity will not exceed 1 pF. Assuming this value, a PCB temperature variation of ±10 °C and a temperature coefficient of the dielectric constant of a FR-4 substrate of 400 ppm/°C, the variation of *C_eP_* is in the range of ±4 fF. Analyzing in Equation (13) the worst case for this contribution from the term associated with ∆Ce/Ce and the components and parameters used in the experiments, the result is a distance error in the range of ±40 µm. This error is negligible with respect to the rest of the contributions, *C_eP_* is therefore excluded from the analysis hereafter.

### 2.3. Relation between Rf and SNR

Some of the previous arguments relied on the assumption that the SNR in a TIA improves with increasing Rf. This can be demonstrated by obtaining the expressions for the contributions of the photodiode signal I_PH_ and the noise of the photodiode and amplifier, depicted in Equation (6). In (6) it is assumed that the common-mode input resistance of the OA and the equivalent shunt resistance of the photodiode are much higher than *Rf* and that the open-loop gain of the OA is ideal. These approximations are completely acceptable for the noise study and enable obtaining expressions that are more compact and simpler to interpret without loss of generality [18]. *i_n_* and *v_n_* represent the noise current and voltage spectral densities of the OA, respectively, I_PH_ is the photocurrent generated by the photodiode, *i_nPH_* the noise current of the photodiode, T the circuit temperature, *K* the Boltzmann constant for thermal noise (1.38 × 10^−23^ J/K) and *Ci* = *C_CM_* + *C_PH_* the sum of the common-mode input capacitance of the OA and the equivalent capacitance of the photodiode.

(6) $${\left|Vo{|}_{I_{PH}}\right|}^{2}={\left|I_{PH}\right|}^{2}{\left|\frac{Rf}{1+jwRfCf}\right|}^{2}\;{\left|Vo{|}_{Noise}\right|}^{2}=\left(i_{n}^{2}+i_{nPH}^{2}+\frac{4KT}{Rf}\right){\left|\frac{Rf}{1+jwRfCf}\right|}^{2}+v_{n}^{2}{\left|\frac{1+jwRf\left(Ci+Cf\right)}{1+jwRfCf}\right|}^{2}\;{\left|\frac{Vo{|}_{I_{PH}}}{Vo{|}_{Noise}}\right|}^{2}=\frac{{\left|I_{PH}\right|}^{2}}{\left(i_{n}^{2}+i_{nPH}^{2}+\frac{4KT}{Rf}\right)+v_{n}^{2}{\left|\frac{1+jwRf\left(Ci+Cf\right)}{Rf}\right|}^{2}}=\frac{{\left|I_{PH}\right|}^{2}}{i_{n}^{2}+i_{nPH}^{2}+\frac{4KT}{Rf}+\frac{v_{n}^{2}}{Rf^{2}}+w^{2}{\left(Ci+Cf\right)}^{2}v_{n}^{2}}$$

As deduced from (6), SNR worsens if the photodiode capacitance *C_PH_* increases (as *Ci* = *C_CM_* + *C_PH_* and the voltage noise term increases). This leads to select low noise voltage OAs, as this is the dominant contribution as opposed to the OA noise current [18,20]. Additionally, according to (6), increasing the value of *Rf* decreases the contribution of the OA noise voltage and *Rf* thermal noise current, hence increasing the SNR. We note that the maximum value of *Rf* is limited by the expected Δ*C_FP_* according to Equation (5). On the other hand, the SNR increases if *Cf* is reduced. Conversely, reducing *Cf* increases the cut-off frequency of the system, moving away from the condition *f* = *f_C_* that allows maximizing the value of Rf obtained by limiting the error generated by variations of the parasitic capacitance *C_FP_* (see Equation (5)). It is, therefore, necessary to determine whether it is convenient to seek the condition *f* = *f_C_* to maximize *Rf* and thus minimize part of the contributions of the denominator of the SNR expression, or focus on reducing *Cf* (*f_C_* > *f*), which implies reducing *Rf* to limit the contribution of *C_FP_*. To resolve this trade-off, the relative noise contribution of the different sources involved must be analyzed. This requires particularizing the data for a given application and, as we are focusing on the OA, studying the denominator terms of Equation (6) without including the noise contribution of the photodiode. 

These terms are represented by *I_on1_* in Equation (7):

(7) $$I_{on1}^{2}\left(Rf,Cf,v_{n},Ci\right)=i_{n}^{2}+\frac{4KT}{Rf}+\frac{v_{n}^{2}}{Rf^{2}}+w^{2}{\left(Ci+Cf\right)}^{2}v_{n}^{2}$$

In typical high-BW applications, *Cf* is significantly lower than *Ci*. Upon inspection of Equation (7), one can see that the noise current is not very sensitive to variations of *Cf*. In these cases, the design condition *f* = *f_C_* allows minimizing the impact of the stability of the parasitic capacitance *C_FP_* and therefore using a higher value of *Rf* thus maximizing the SNR.

### 2.4. Phase Stability Considering Non-Ideal OA Parameters

The contributions of the real parameters of the OA on the phase stability should also be investigated. Analyzing the circuit in Figure 3, Equation (9) is obtained, where *w_OA_* is the cut-off frequency of the open-loop gain of the OA, *Ao* the open-loop DC gain of the OA, and *s_OA_*, *s_C_* and *s_X_* are defined as:

(8) $$\begin{array}{c} s_{OA}=w_{OA}\\ s_{C}=w_{C}=\frac{1}{Rf\cdot Cf}\\ s_{X}=w_{X}=\frac{1}{Rf\cdot \left(Cf+Ce\right)} \end{array}$$

(9) $$TI\left(s\right)=\frac{Vo\left(s\right)}{I_{PH}\left(s\right)}=\frac{-Rf}{\left(1+\frac{1}{Ao}+\frac{Rf}{Ao\cdot Re}\right)+\left(\frac{1}{s_{C}}+\frac{1}{Ao\cdot s_{C}}+\frac{1}{Ao\cdot s_{OA}}+\frac{Rf}{s_{C}\cdot Re\cdot Ao}+\frac{Rf}{s_{OA}\cdot Re\cdot Ao}\right)s+\frac{1}{s_{X}\cdot s_{OA}\cdot Ao}s^{2}}≅\;≅\frac{-Rf}{\left(1+\frac{1}{Ao}+\frac{Rf}{Ao\cdot Re}\right)+\left(\frac{1}{s_{C}}+\frac{1}{Ao\cdot s_{OA}}+\frac{Rf}{s_{OA}\cdot Re\cdot Ao}\right)s+\frac{1+\frac{Ce}{Cf}}{s_{C}\cdot s_{OA}\cdot Ao}s^{2}}$$

assuming that *s_OA_* << *s_C_*_,_ which applies to practical cases. Additionally, *s_C_* is much more stable, hence its increments are smaller and its contribution to incremental phase deviations is negligible compared to those caused by *s_OA_*. The phase introduced by the system is obtained in Equation (10):

(10) $$\alpha \left(w,w_{C}\right)=∠TI\left(s=jw\right)=\pi -arctan\left(F\left(w,w_{C}\right)\right)$$

where *F*(*w,w_C_*) is:

(11) $$F\left(w,w_{C}\right)=\frac{\frac{w}{w_{C}}\left(1+\frac{w_{C}}{Ao\cdot w_{OA}}+\frac{w_{C}}{Ao\cdot w_{OA}}\frac{Rf}{Re}\right)}{1+\frac{1}{Ao}\left[1+\frac{Rf}{Re}-\frac{w^{2}}{w_{C}\cdot w_{OA}}\left(1+\frac{Ce}{Cf}\right)\right]}≅\;≅\frac{w}{w_{C}}\left[1+\frac{1}{Ao\cdot w_{OA}}\left[w_{C}\left(1+\frac{Rf}{Re}\right)+\frac{w^{2}}{w_{C}}\left(1+\frac{Ce}{Cf}\right)\right]\right]=F\left(w,w_{C}\right){|}_{Ideal}\left(1+\frac{\Delta F\left(w,w_{C}\right){|}_{Ideal}}{F\left(w,w_{C}\right){|}_{Ideal}}\right)$$

In (11), the approximations $\left|\frac{1}{Ao}\left[1+\frac{Rf}{Re}-\frac{w^{2}}{w_{C}\cdot w_{OA}}\left(1+\frac{Ce}{Cf}\right)\right]\right|\ll 1$ and $w_{C}\ll w_{OA}$ were used in order to obtain a simplified expression that facilitates the analytical study. Equation (12) represents the phase difference with respect to that obtained assuming an ideal OA with infinite open-loop gain (*F*(*w,w_C_*)|*_Ideal_* = *w/w_C_*), i.e., the additional phase contributions due to the non-ideal OA parameters are:

(12) $$\Delta \alpha \left(w,w_{C}\right)≅\frac{d\alpha \left(w,w_{C}\right)}{dF\left(w,w_{C}\right)}\Delta F\left(w,w_{C}\right)=-\frac{1}{1+{\left(\frac{w}{w_{C}}\right)}^{2}}\frac{w}{w_{C}}\frac{1}{Ao\cdot w_{OA}}\left[w_{C}\left(1+\frac{Rf}{Re}\right)+\frac{w^{2}}{w_{C}}\left(1+\frac{Ce}{Cf}\right)\right]$$

The stability (deviations) of the measured phase and its corresponding distance (Δα and Δd respectively) are shown in Equation (13). This has been obtained by calculating the variation Δα in Equation (12) due to the OA parameters, where the frequency *f* is used as a variable, in order to include in the result the GBWP (gain-bandwidth product) routinely provided by OA manufacturers.

(13) $$\Delta \alpha \left(f,f_{C}\right)≅\frac{1}{1+{\left(\frac{f}{f_{C}}\right)}^{2}}\frac{1}{GBWP}\left[f\left(\left(1+\frac{Rf}{Re}\right)+\frac{f^{2}}{f_{C}^{2}}\left(1+\frac{Ce}{Cf}\right)\right)\frac{\Delta GBWP}{GBWP}+f\frac{Rf}{Re}\frac{\Delta Re}{Re}-\frac{f^{3}}{f_{C}^{2}}\frac{Ce}{Cf}\frac{\Delta Ce}{Ce}\right]=\;=\frac{f}{1+{\left(\frac{f}{f_{C}}\right)}^{2}}\frac{1}{GBWP}\left[\left(\left(1+\frac{Rf}{Re}\right)+\frac{f^{2}}{f_{C}^{2}}\left(1+\frac{Ce}{Cf}\right)\right)\frac{\Delta GBWP}{GBWP}+\frac{Rf}{Re}\frac{\Delta Re}{Re}-\frac{f^{2}}{f_{C}^{2}}\frac{Ce}{Cf}\frac{\Delta Ce}{Ce}\right]\;\Delta d\left(f,f_{C}\right)=\frac{c}{2\pi f}\Delta \alpha \left(f,f_{C}\right)=\frac{c}{1+{\left(\frac{f}{f_{C}}\right)}^{2}}\frac{1}{2\cdot \pi \cdot GBWP}\left[\left(\left(1+\frac{Rf}{Re}\right)+\frac{f^{2}}{f_{C}^{2}}\left(1+\frac{Ce}{Cf}\right)\right)\frac{\Delta GBWP}{GBWP}+\frac{Rf}{Re}\frac{\Delta Re}{Re}-\frac{f^{2}}{f_{C}^{2}}\frac{Ce}{Cf}\frac{\Delta Ce}{Ce}\right]$$

According to Equation (13), for a given *f_C_* the phase stability improves by increasing *Cf* and reducing *Rf*, which penalizes the SNR of the system. Similarly, it is of interest that the parasitic elements *Re* and *Ce* tend to their ideal value, that is, *Re* = ∞ and *Ce* = 0. As for the values of *Rf* and *Cf*, the trade-off between SNR and phase stability appears again, as was the case when considering the effect of variations of the parasitic feedback capacitance *C_FP_*. Regarding the influence of the *f*/*f_C_* ratio on the distance stability deduced in Equation (13), the terms associated with *Rf*/*Re* introduce a low-pass frequency weighting, while those associated with *Ce*/*Cf* introduce a high-pass frequency weighting. If we assume that *f_C_* ≥ *f*, the low-pass weighting applied to the terms associated with *Rf*/*Re* can be between 1 for *f* << *f_C_* and 0.5 for *f* = *f_C_*, while the high-pass weighting applied to the terms associated with *Ce*/*Cf* can be between 0.5 for *f* = *f_C_* and 0 for *f* << *f_C_*. Taking the ratio *f* = *f_C_* gives the same weighting of 0.5 to all the contributions, resulting in Equation (14), which enables a direct comparison of the relative relevance of each of the contributions. Again, for phase and distance deviations, Δ*α* and Δ*d*, it follows:

(14) $$\Delta \alpha \left(f=f_{C}\right)≅\frac{1}{2}\frac{f}{GBWP}\left[\left(2+\frac{Rf}{Re}+\frac{Ce}{Cf}\right)\frac{\Delta GBWP}{GBWP}+\frac{Rf}{Re}\frac{\Delta Re}{Re}-\frac{Ce}{Cf}\frac{\Delta Ce}{Ce}\right]\;\Delta d\left(f=f_{C}\right)=\frac{c}{2\pi f}\Delta \alpha \left(f=f_{C}\right)≅\frac{c}{4\cdot \pi \cdot GBWP}\left[\left(2+\frac{Rf}{Re}+\frac{Ce}{Cf}\right)\frac{\Delta GBWP}{GBWP}+\frac{Rf}{Re}\frac{\Delta Re}{Re}-\frac{Ce}{Cf}\frac{\Delta Ce}{Ce}\right]$$

Considering Equations (13) and (14), it is possible to improve stability by increasing *Cf* and reducing *Rf*, at the cost of penalizing the SNR. This improvement; however, is practically limited. Even though *Re* >> *Rf* and *Ce* << *Cf* are satisfied, Equations (13) and (14) yield Equation (15), where the error due to the GBWP is still determinant:

(15) $$\left|\Delta d(f,f_{C}\right|\geq \left|\frac{c}{2\cdot \pi \cdot GBWP}\frac{\Delta GBWP}{GBWP}\right|$$

In effect, if in this expression we make GBWP = 4 GHz (currently around the highest available values for commercially available OAs) and a drift of this parameter of ±10% (easily surpassed in a real OA subject to temperature ranges of some tens of °C) the deviation in the distance measurement can reach ±1.2 mm. Please note that this was deduced for the best case, under very favorable conditions, which are hardly realizable. Therefore, in real applications such as the one shown in Figure 2, millimeter-level stabilities are challenging (though possible) to achieve due to the OA contribution.

**Figure 5:**
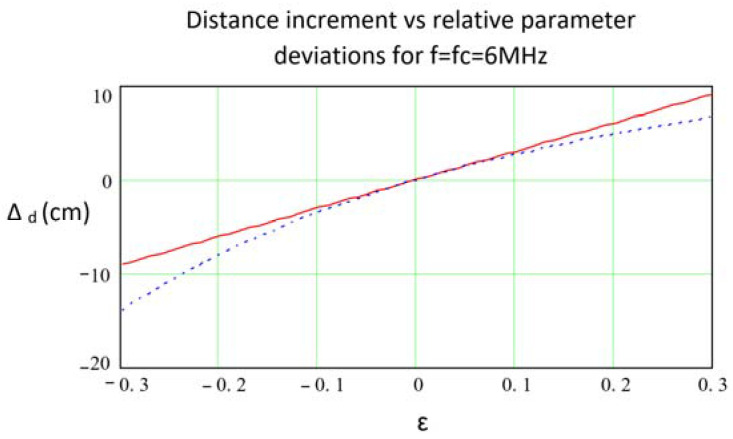
Phase stability computed from Equation (9) (blue/dashed) and from the approximated expression in Equation (14) (red/solid) as a function of *ε* = Δ*GBWP*/*GBWP* = Δ*Re*/*Re* = −Δ*Ce*/*Ce*, obtained for an OA OPA847, *Rf* = 22 KΩ, *Cf* = 1.2 pF, *Ce* = 18.7 pF and *f* = *f_C_* = 6 MHz.

Figure 5 shows, for the data indicated therein, the stability in the distance measurement provided by the approximation obtained with Equation (14) together with that obtained from the non-approximated expression in Equation (9). Both were evaluated considering the worst case of the arithmetic sum of the contributions from different sources of instability and assumed Δ*GBWP*/*GBWP* = Δ*Re*/*Re* = −Δ*Ce*/*Ce* = *ε*. As can be seen, the approximations reflected in Equation (14) and consequently (15) are practically useful for the system design, since they clearly reflect the contribution of each of the parameters while providing an estimation very close to that of the original functions.

## 3. Summary of Design Considerations

We recall herein the concepts addressed in detail in the previous section. In Table 1 they are also condensed and readable at first glance (symbols “↑” and “↓” indicate respectively the convenience of increasing or reducing certain parameter value). A last point also includes the most relevant design guidelines.

The analysis developed is summarized as follows:

- **Problem approach.** The presented analysis focuses on the phase shift stability (defined as phase deviation) due to the thermal drifts of the component parameters demonstrating its trade-off with SNR, and its dependency upon the TIA *Rf*-*Cf* feedback network. Together with *Rf* and *Cf*, the elements and parameters involved are (see Figure 3): the OA real parameters (*GBWP* and input impedance), photodiode parameters (equivalent impedance), circuit board parasitic capacitances, and relation between the modulating frequency f and the −3 dB cutoff frequency *fc*.
- **TIA transfer function. Cutoff frequency and *Rf*, *Cf* roles.** From the phase expression of the TIA transfer function and considering first an ideal OA, it would be convenient to increase the −3 dB cutoff frequency f_C_ as much as possible i.e.: *f_C_* >> *f* (with *f* being the signal operating frequency). This means reducing the value of *Rf* and/or *Cf* (so as to reduce the product *Rf*⋅*Cf*). This is addressed in Equations (1)–(3).
- **PCB parasitic capacitance (*C_FP_*).** When reducing *Cf* (to increase *fc*) the effect of the printed circuit board (PCB) parasitic capacitance *C_FP_* in parallel with *Cf* in the TIA feedback loop (circuit of Figure 3) becomes significant. Additionally, from Equation (3), reducing Rf lowers the effect of *C_FP_* increment.
  - **SNR implications.** Conversely, SNR is improved by increasing the value of *Rf* (SNR is further addressed in detail in Equations (6) and (7)). At the same time, as deduced from Equation (5), the maximum value for Rf in order to preserve a certain distance error under the variations in *C_FP_* is held at the condition *f* = *fc* (assuming sinusoidal modulation within the bandwidth).
  - **Design condition f = fc trade-off.** The design condition *f* = *fc* (i.e.: operating frequency equal to the −3 dB cutoff frequency) implies the following trade-off: on the one hand, it is the worst choice regarding *Cf* and *Rf* variations (as shown in Figure 4 and Equations (1)–(3) pointed out above in ii.). On the other hand, *f* = *fc* is the best solution regarding *C_FP_* variations and SNR (Equation (5)).
- **SNR, Rf and Cf**. Noise contributions and SNR are analyzed in Equations (6) and (7). It is shown that increasing *Rf* reduces noise (note that reducing *Rf* was found convenient for improving stability).
- **Real OA**. Finally, when including the parameters of a real OA in the analysis (GBWP and input impedance), again the same trade-off between SNR and stability as stated in iii.b is found. The analysis is developed through Equations (9)–(15), where some valid simplifications (under typical conditions) were imposed. From Equation (14) the theoretical *Rf* and *Cf* optimal values could be deduced, although this is not a practical solution due to the uncertainties of the real component values. In practice, a good solution is again to increase *Cf* and reduce *Rf*, at the assumable cost of worsening SNR.

### Practical Design Guidelines

- After analyzing all contributions in detail, for certain applications, and depending on the component’s real parameters, the design condition *f* = *fc* can be a good trade-off solution. Nevertheless, this will depend on each case which must be individually analyzed.
- Increasing the value of *fc* is a valid solution in order to achieve sub-mm stability under variations of *Rf* and *Cf* due to thermal drift. This means reducing *Rf* and/or *Cf* nominal design values.
- The effect of the parasitic capacitance *C_FP_* can be also minimized by reducing *Rf*.
- In both cases (stated in two previous points) reducing *Rf* is at the cost of worsening SNR. In many typical applications, as in the case presented here, this SNR reduction is perfectly assumable.
- Regarding OA, it must have a high *GBWP*, low input capacitance and high input resistance, as well as low thermal drift of these parameters. Among the set off all OA parameters, these are the relevant ones regarding phase stability.
- In this regard, Equation (15) is a good design aid for selecting the OA, as it defines the best possible stability given a certain OA.

## 4. Results

The analysis carried out in the previous sections is applied herein to a real distance measurement application. We test the stability of an infrared (IR) distance measuring system against variations due to thermal drifts. We first address the design considerations studied throughout the paper, now applied to the specific characteristics of this case, and we show the real measurement results, which are also compared with the theoretical predictions.

**Figure 6:**
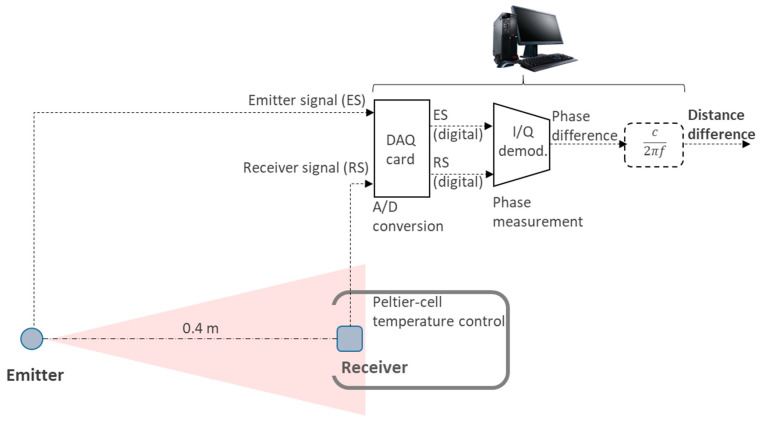
Test setup for the validation of the case study.

The setup under test is an IR link (emitter and receiver), which forms part of the indoor localization system presented in [7,8]. This setup is shown in Figure 6. We use an infrared LED with a 6 MHz sinusoidal intensity-modulation and a silicon PIN photodiode (Advanced Photonix SD100-11-31-221), inverse-biased with a very stable 5 V reference, a capacitance C_PH_ ≈ 15 pF and a shunt resistance greater than 20 MΩ. The TIA is built with the OA OPA847ID with ±2.5 V voltage supply. 

To control the temperature, the photodiode and TIA are placed in a metallic box with a control system based on a Peltier cell and a temperature sensor (note this thermal stability control is just needed to carry out tests, not in real performance). The phase between emitted and received signals is measured with a phase meter based on I/Q demodulation. Further conversion to distance is straightforward knowing the operating frequency (6 MHz). As can be seen, the emitted and received signals are digitized with a data acquisition card, and thus processing stages are implemented in a PC [7,8,24]. A 0.4 m distance between emitter and receiver was set so that SNR does not affect the phase deviation measurement caused by parameter drifts, which is the target of the test conducted here and, consistently, the setup is intended to characterize this effect. We can ascertain by design that, under these setup conditions, noise error has negligible levels compared with thermal drift deviations. Therefore, drift errors can be measured without being masked by noise, as can also be observed further in the measurement plots, where noise introduces very low dispersion. Under real conditions, i.e., larger emitter-target distances and/or angles (deviation from the emitter central axis), thermal drifts will remain the same independently from signal or noise levels. 

In the tests we apply a ±10 °C temperature variation. This 20 °C interval covers the expected temperature range in a regular indoor environment (wider range could be tested, if necessary, proceeding the same way as exposed here). As a requirement, we impose a maximum distance standard deviation of ±1 cm due to delay time (or, equivalently, phase) deviations.

### 4.1. Design Calculations

As discussed in the previous sections, the condition f = fc is imposed, thus maximizing the value of *Rf* for a given parasitic capacitance *C_FP_*, and balancing the contributions to instability due to Re and Ce with the same weight. 

As explained from Equation (4), high stability of *Rf* and *Cf* is needed. High-thermal-stability resistors are available, but this is not the case with capacitors, thus making them the most critical component. Working with a thermally stable ceramic NPO capacitor *Cf* with thermal coefficient of ±30 ppm/°C and a resistor with a ±10 ppm/°C thermal coefficient results in a joint thermal drift of ±31.6 ppm/°C (Equation (3)). From Figure 4, it can be seen that for a ±10 °C temperature swing and *f* = *fc* = 6 MHz, the distance standard deviation due to this concept is an assumable value of ±0.126 cm. 

As for the effect of the PCB parasitic capacitance *C_FP_*, with a conservative criterion we assume the following values: *C_FP_* = 0.2 pF, TC(*C_FP_*) = 300 ppm/°C and Δ*T* = ±10 °C, resulting in Δ*C_FP_* = ±0.6 × 10^−3^ pF. A limit value for *Rf* of 111 KΩ guarantees a distance uncertainty (standard deviation) below ±1 cm according to Equation (5). Aiming at keeping a margin for the rest of contributions, *Rf* = 22 KΩ is chosen for the experiments conducted. With this value the distance standard deviation due to this contribution is kept below ±0.2 cm. Additionally, with this *Rf* value, together with a value for the capacitance *Cf* = 1 pF, the condition *fc* ≈ 6 MHz is fulfilled.

Four candidate OAs are shown in Table 2, all of them with low noise and high GBWP (the first three have bipolar input technology while the fourth one has FET technology). These four candidates were chosen so that each one has better performance than the others with respect to one (or more) of the relevant parameters. In this regard, integrated gain OAs could also be considered as a possible solution, but usually there is lack of information about the parameters of interest in this type of OAs (namely GPBW and input impedance). Besides, they do not allow for flexible design, i.e., *Rf* optimal tuning. Nevertheless, under certain conditions (low integrated *Rf*), they could be taken into account as reflected in the conclusions section. The best OA choice is reached after analyzing Table 2, Table 3 and Table 4 jointly. The arguments to choose the OPA847ID are exposed below Table 4. 

With the design values stated before, the contributions to the distance deviation determined by Equation (14) are shown in Table 3 for all OAs considered. The condition *f* = *fc* still applies, *Ce* = *Cin* + *C_PH_* and *Re* = Rin//20 MΩ.

According to Table 3, the only way to force the effects of the OA to low values is by using devices with a very high GBWP. To quantify the distance error we make two possible assumptions on the parameters involved (GBWP, Re and Ce). If, for instance, we take the relative deviations of GBWP, Re and Ce as zero-mean random variables with 15% standard deviation, the standard deviation of the measured distance is as reflected in the first column of Table 4. Or, alternatively, note that deviations due to thermal coefficients may have a fixed sign deviation, thus, we can consider deviations as signed maximum deviations and compute the error as the worst case (arithmetic sum of the different contribution moduli). This is shown in the second column of Table 4. Since quite often the nature of the error parameters provided by manufacturers is not explicitly defined, both assumptions are of interest. In both cases, as seen in Table 4, the error is at the cm level, clearly lower in the case of first and third OAs considered.

To choose the best OA, first note that, since the signal is modulated and the useful information is contained in the AC component, bias current contribution is not relevant (as it just introduces a DC deviation), as long as it does not make the output saturate. As deduced from previous sections, we are mainly interested in high GBWP (for low circuit propagation time variations). Besides, as in our design we have a high photodiode input capacitance, the OA voltage noise becomes a dominant factor. For this reason, a bipolar input OA is a convenient choice (first three ones in Table 2). From Table 4, OPA847ID and THS4021CD are the two best OA candidates. In this work, the OPA847ID was chosen for the tests, as it shows better noise features. The FET-input OA was also included in Table 2 (THS4021CD) to show a wider initial fan of possible choices. This OA has high input impedance (which is a good feature as its effect on phase variations is negligible) but, in contrast, also higher noise. It also shows low input bias current although, as explained, it is not a big concern in this case, while noise voltage is. 

As discussed in the previous sections, the OA error, as well as the TC(*Rf*), TC(*Cf*) and ΔC_FP_ ones, is reduced by reducing *Rf*. As can be seen in Table 4, a standard deviation of the thermal drifts of *Re*, *Ce* y GBWP below ±5% is needed in order to achieve 1 cm standard deviation in the measured distance. 

In practice, one of the most severe design handicaps is the lack of information provided by manufacturers about thermal effects of the OA and photodiode parameters. Moreover, such thermal drifts can reach high values [25], as demonstrated in the next subsection with measurement results, where it will be seen that real deviations can be greater than the ±5% margin defined above even under moderate temperature variations.

Consequently, reducing *Rf* and/or increasing *Cf* will be needed in order to keep the phase (and distance) stability within the required margins. 

**Figure 7:**
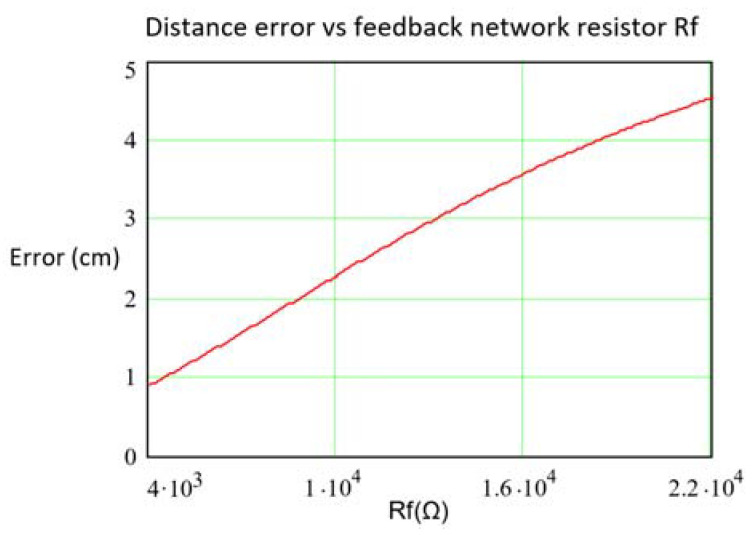
Maximum distance error modulus (cm) deduced from Equation (13) as a function of *Rf* (Ω), for the OA OPA847ID with *Cf* = 1.2 pF and *Ce* = 18.7 pF. Variations of *Ce*, *Re* and GBWP of ±15%.

**Figure 8:**
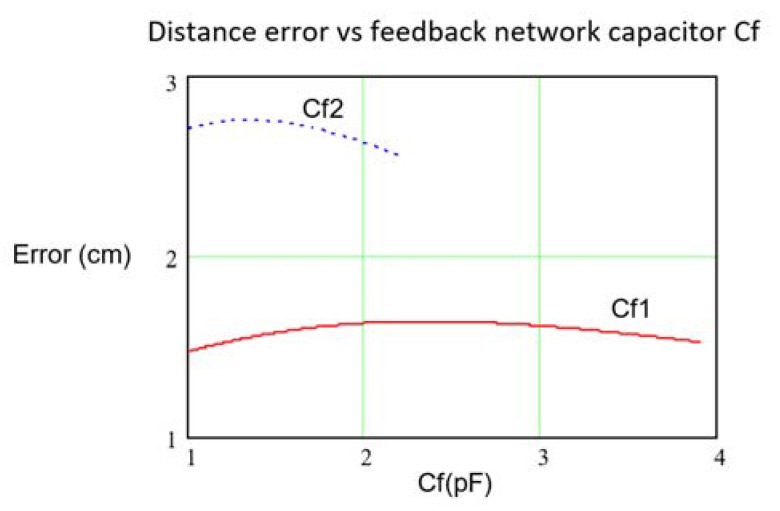
Maximum distance error modulus (cm) deduced from Equation (13) as a function of *Cf* (*Cf*1 up to 3.9 pF and *Cf*2 up to 2.2 pF), for the OPA847ID with *Rf*1 = 6.8 KΩ, Rf2 = 12 KΩ and *Ce* = 18.7 pF. Variations of *Ce*, *Re* and GBWP of ±15%.

To quantify the effect of *Rf* and *Cf* on the distance error, the shape of the distance error given by Equation (13), keeping *Cf* = 1.2 pF and reducing the value of *Rf* from 22 KΩ to 4 KΩ (at *Rf* = 22 KΩ the condition *f_C_* ≈ 6 MHz=f holds) can be seen in Figure 7. As can be observed, the stability improvement as Rf is lowered is quite meaningful. In Figure 8, the distance error for two *Rf* values (6.8 KΩ and 12 KΩ) as a function of *Cf*, is depicted. As seen, to achieve *f_C_* ≈ 6 MHz the pair of valid values (*Rf*, *Cf*) are (6.8 KΩ, 3.9 pF) and (12 KΩ, 2.2 pF), which define, therefore, the maximum values of Cf in both situations. This behavior can be explained by Equation (13), where the terms with *Rf* weight the low pass response while the terms with *Cf* weight the high pass response. 

Regarding noise, as also explained throughout the paper, it worsens under conditions that lead to an improvement in stability, i.e., reducing *Rf* and/or increasing *Cf*, also reflected in Equation (7). In Table 5 all the contributions to uncertainty studied in this work are included for different *Rf* values. In all cases the resultant TIA is stable, showing an underdamped response in which the BW is practically independent of the photodiode capacitance, and it is determined by the RC feedback network (*Rf* and *Cf*) [14,20]. As observed in this Table, with the parameters shown, values of *Rf* around 3.9 KΩ or smaller are needed to achieve *a* ± 1 cm stability margin. This is an extremely low value in typical applications with BW in the order of some MHz but, as will also be demonstrated with real measurements, these *Rf* values are needed to guarantee the required phase stability. Besides, the increment in the current noise (29% with respect to the minimum, which is 2.57 pA/√Hz) as a consequence of lowering *Rf* is not severe and is perfectly assumable.

### 4.2. Measurements

**Figure 9:**
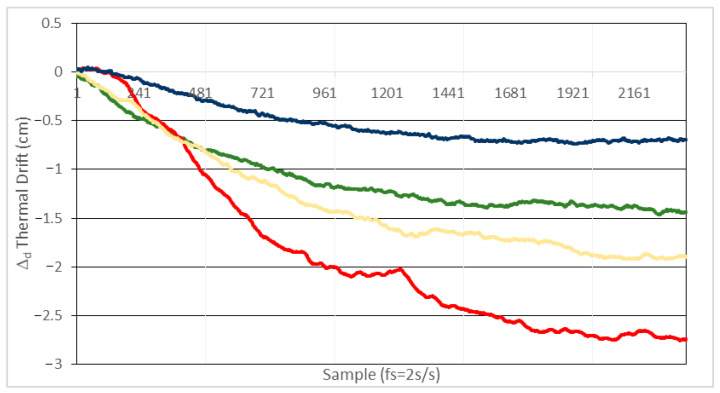
Distance error due to phase thermal drift with Cf = 1.2 pF and temperature increment from 25 °C to 35 °C (+10 °C). Blue line: Rf = 3.9 KΩ, green line: Rf = 6.8 KΩ, orange line: Rf = 12 KΩ and red line: Rf = 22 KΩ.

**Figure 10:**
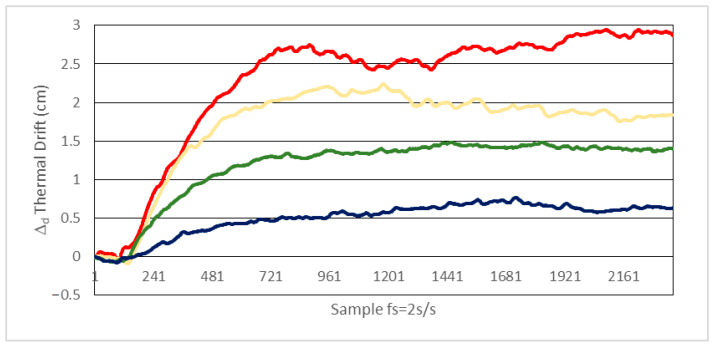
Distance error due to phase thermal drift with *Cf* = 1.2 pF and temperature increment from 25 °C to 15 °C (−10 °C). Blue line: *Rf* = 3.9 KΩ, green line: *Rf* = 6.8 KΩ, orange line: *Rf* = 12 KΩ and red line: *Rf* = 22 KΩ.

We conducted two experimental tests with the setup shown in Figure 6:

- In a first test, after the system reaches the permanent thermal regime at 25 °C, it is subjected to a +10 °C temperature increment. In Figure 9 the distance error caused by phase thermal drift is depicted, for the different values of Rf shown in Table 5.
- In a second test, starting at the permanent thermal regime at 25 °C, the system is subjected to a −10 °C temperature increment. The errors due to thermal drifts are depicted in Figure 10.

As observed in Figure 9 and Figure 10, in order to keep the distance error within ±1 cm, the temperature swing must be within a ±10 °C interval for values of *Rf* around 3.9 KΩ or smaller. For the same value of *Rf* the results are very similar for positive and negative temperature deviations, delivering a distance deviation within ±0.8 cm (equivalent to ±1 mrad phase deviation at 6 MHz) for *Rf* = 3.9 KΩ and temperature increments of ±10 °C. As mentioned before, the temperature range tested covers the expected variations in an indoor environment under normal conditions. If a wider interval is considered, the same type of tests can easily be carried out. In such a case, regarding possible phase deviation being masked by noise, we can get an idea about the expected order of magnitude of this effect: for a temperature variation of, for instance, up to 85 °C, the increment in the noise contribution would be below 10%, due to thermal noise of Rf and the OA noise characteristics, extracted from the datasheet.

From the results of Figure 9 and Figure 10 we conclude that the results are consistent with the theoretical predictions. Considering the values in Table 5 altogether, we can ascertain that thermal variations of the parameters involved in Equation (13) must necessarily be high. Determining every individual contribution of each parameter to the global error is not straightforward, as the thermal coefficients of the OA and photodiode parameters of interest are not provided by the manufacturers. 

At this point, two different strategies can be determined: one is to design ad hoc experiments to measure the thermal drift of each parameter. The other consists of measuring a global circuit thermal drift with one single experiment. The second approach, chosen in this work, is more practical, simple and reliable. Furthermore, individual specific tests on every parameter could even not be appropriate to determine the global circuit thermal deviation. 

In this way, we design the TIA according to the conclusions derived from this work and subject the entire system to stability tests, reducing the value of Rf up to a value at which an appropriate thermal stability is reached. High Rf values are customarily chosen in photodiode conditioning circuits, while in this work, it was demonstrated that it may be convenient to proceed otherwise, i.e., taking low Rf values. Furthermore, even when low Rf values are selected, it is usually for BW increasing purposes, whereas here the goal is different: enhancing stability under thermal drifts.

## 5. Conclusions

In this work, we addressed the basic concepts in the design of a photodiode signal conditioning transimpedance amplifier (TIA) in distance measurement systems based on phase-shift measurement, when high stability in the time delay (or phase) is needed. The influence of the operational amplifier (OA) noise was also addressed, as well as its implications in the design values of the components under the strong trade-off between measured distance stability and SNR. A thorough analysis was carried out throughout Section 2.1, Section 2.2, Section 2.3 and Section 2.4 and, for easier reading, a synthesis of the main design concepts and a summary of the relevant design guidelines was presented in Section 3. The last paragraphs of the measurements section are also of particular interest in this regard.

The theoretical analysis and the results demonstrate that an OA with high gain-bandwidth product and high open loop input impedance is needed. These parameters must also have high thermal stability. On the other hand, a critical design aspect is the choice of the TIA feedback resistor Rf, affecting dramatically the thermal stability of the delay time. Unlike typical designs, focused on minimizing the amplifier noise effects but not the stability against thermal drifts, which may introduce unacceptable errors in the distance measurement, in this design it is necessary to use unusual low Rf values. With the design guidelines proposed in this work, high stability (hence low thermal deviation errors) is achieved at the expense of an increment in the OA noise. This effect (noise increment) reduces SNR and consequently precision, but its impact on the final error is lower than the benefits for stability and remains within assumable levels. 

As regards the OA choice, the relation stated in Equation (15) provides a powerful design tool as it defines the best achievable stability for a specific OA used in the design. Integrated gain OAs can also be considered in future as an interesting solution as long as they have low Rf values to meet the design requirement concluded in this work.

The effect of the printed circuit board (PCB) parasitic capacitances is another key aspect studied in this work. It has been demonstrated that PCB design oriented to reduce the parasitic capacitance in the OA feedback network mitigates its effect on the stability. 

Regarding the deviation caused by thermal stability of the TIA RC feedback network (Rf and Cf), it can be reduced to negligible values with conventional components. The TIA phase stability due to this RC network is improved with low values of Rf, in order to increase the cutoff frequency, determined by the OA parameter contributions to thermal instability. 

The designed circuit was tested by conducting two distance measurement experiments, showing stability results within ±1 cm for temperature variations in a ±10 °C range, which corresponds to phase deviations within ±1.25 mrad with a modulation frequency of 6 MHz. As seen in the results, without a careful design, the instability of the measured delay time or phase due to thermal drifts can cause unacceptable errors. 

Information regarding key design parameters (namely thermal coefficients) is rarely provided by manufacturers and, when available, it is not precise enough to extract valid values. An approach based on a global single test to obtain the entirety of the circuit deviations due to thermal drifts has been applied, as opposed to a less reliable and efficient strategy based on individual ad hoc tests for each parameter. In any case, having this detailed information available would be a great aid to select an appropriate OA.

We conclude that results are consistent with the theoretical predictions. Additionally, by contrasting the theoretical model with the measurements, high thermal drifts of the OA parameters of interest have been observed. Consequently, without a careful design addressing all possible contributions, the error can rise to unacceptable levels due to thermal effects. 

## Figures and Tables

**Table 1:** Effect of the circuit parameters on SNR and distance stability. *Re* = *R_CM_*//*R_DM_*//*R_PH_*, *Ce* = *C_CM_* + *C_DM_* + *C_PH_* (see Figure 3).

**Improve** **SNR**	**General**	**Supposing *Cf* << *Ci* = *C_CM_* + *C_PH_* (typical in high BW systems)**			
	*Rf*↑ y *Cf*↓	Rf↑. Effect of *Cf* is negligible (minimum value is determined by TIA stability to avoid oscillations)			
**Improve** ***S_d_***	**Instability**				
	**TC(*Rf*), TC(*Cf*)**	**TC(*C_FP_*)**	**Δ*GBWP*/*GBWP***	**Δ*Re*/*Re***	**Δ*Ce*/*Ce***
	(*f*/*fc*)↑ *fc*↑( *Rf*↓, *Cf*↓)	(*f*/*fc*)↑ *Rf*↓	(*Rf*/*Re*)↓, *Rf*↓, *Re*↑ (*Ce*/*Cf*)*↓, Cf*↑, *Ce*↓ *GBWP*↑ (*f*/*fc*) Effect depends on value of: *Rf*/*Re* and *Ce*/*Cf*	(*Rf*/*Re*)↓, *Rf*↓, *Re*↑ *GBWP*↑ (*f*/*fc*)↑	(*Ce*/*Cf*)↓, *Cf*↑, *Ce*↓ *GBWP*↑ (*f*/*fc*)↓

**Table 2:** Commercial OA characteristic parameters (typical values). Columns from left to right: input noise voltage, input current noise, gain-bandwidth product, input capacitance, input resistance.

	Vn(nV/√Hz)	In(pA/√Hz)	GBWP(GHz)	Cin(pF)	Rin(KΩ)
OPA847ID	0.85	2.5	3.9	3.7	2.7
THS4031D	1.6	1.2	0.2	1.5	2000
THS4021CD	1.5	2	3.5	1.5	1000
ADA4817-1	4	2.5·10^−6^	0.4	1.4	5·10^8^

**Table 3:** Contributions to distance stability due to different OAs according to Equation (14). *Cf* includes the value of C_FP_ = 0.2 pF (*Cf* = 1 pF + *C_F_*_P_ = 1.2 pF) and *Rf* = 22 KΩ.

OPA847ID	$\Delta d\left(cm\right)=15.74\frac{\Delta GBWP}{GBWP}+4.98\frac{\Delta Re}{Re}-9.54\frac{\Delta Ce}{Ce}$
THS4031D	$\Delta d\left(cm\right)=188\frac{\Delta GBWP}{GBWP}+0.13\frac{\Delta Re}{Re}-164\frac{\Delta Ce}{Ce}$
THS4021CD	$\Delta d\left(cm\right)=10.76\frac{\Delta GBWP}{GBWP}+0.015\frac{\Delta Re}{Re}-9.38\frac{\Delta Ce}{Ce}$
ADA4817-1	$\Delta d\left(cm\right)=93.49\frac{\Delta GBWP}{GBWP}+0.007\frac{\Delta Re}{Re}-81.55\frac{\Delta Ce}{Ce}$

**Table 4:** Distance error due to OA (from Table 3). Rf = 22 KΩ, Cf = 1 pF + C_FP_ = 1.2 pF. Left column: contributions are considered zero-mean and σ = 15% random variables. Right column: maximum deviation of error contributions of ±15% (considering maximum signed deviations).

	Distance Error Standard Deviation (cm).	Maximum Error (cm)
OPA847ID	2.86	±4.54
THS4031D	37.3	±52.8
THS4021CD	2.14	±3.03
ADA4817-1	18.6	±26.2

**Table 5:** Errors and noise with OPA847ID. *Cf* includes the value *C_FP_* = 0.2 pF; *Ce* = 18.7 pF.

OPA847ID	−3 dB Cutoff Frequency (MHz). Estimated from Analysis with Figure 3	Maximum Uncertainty (cm) due to OA. Worst Case Arithmetic Sum with ±15% Variations (Equation (13)).	Uncertainty (cm) due to TC(*Rf*) = ±10 ppm/°C + TC(*Cf*) = ±30 ppm/°C and Δ*C_FP_* = 0.6·10^−3^ pF, with Δ*T* = ±10 °C (Equations (4) and (5))	Noise Current (pA/√Hz), Equation (7), for *f* = 6 MHz and *T* = 300K
*Rf* >> 22K for minimum noise (≈ i_n_ of OA)				2.57
*Rf* = 22 K *Cf* = 1.2 pF	6.1	±4.54	±0.16 ± 0.2	2.72
*Rf* = 12 K *Cf* = 1.2 pF	11.5	±2.74	±0.13 ± 0.17	2.84
*Rf* = 6.8 K *Cf* = 1.2 pF	21.1	±1.51	±0.09 ± 0.11	3.02
*Rf* = 3.9 K *Cf* = 1.2 pF	39.6	±0.87	±0.05 ± 0.07	3.31

## Data Availability

Data available on request.

## References

[1] Berkovic G., Shafir E. (2012). Optical methods for distance and displacement measurements. Adv. Opt. Photonics.

[2] Amann M., Bosch T.M., Lescure M., Myllylae R.A., Rioux M. (2001). Laser ranging: A critical review of unusual techniques for distance measurement. Opt. Eng..

[3] McManamon P. (2015). Field Guide to Lidar.

[4] Stone W.C., Juberts M., Dagalakis N., Stone J., Gorman J., Bond P.J., Secretary U., Bement A.L. Performance Analysis of Next-Generation LADAR for Manufacturing, Construction, and Mobility. http://citeseerx.ist.psu.edu/viewdoc/summary?doi=10.1.1.11.2315.

[5] Rüeger J.M. (2012). Electronic Distance Measurement: An Introduction.

[6] Muralikrishnan B., Phillips S., Sawyer D. (2016). Laser trackers for large-scale dimensional metrology: A review. Precis. Eng..

[7] Martin-Gorostiza E., Meca F.J., Galilea J.L.L., Martos-Naya E., Naranjo F.B., Esteban Ó. (2010). Coverage-Mapping Method Based on a Hardware Model for Mobile-Robot Positioning in Intelligent Spaces. IEEE Trans. Instrum. Meas..

[8] Gorostiza E.M., Lázaro Galilea J.L., Meca Meca F.J., Salido Monzú D., Espinosa Zapata F., Pallarés Puerto L. (2011). Infrared Sensor System for Mobile-Robot Positioning in Intelligent Spaces. Sensors.

[9] Hansard M., Lee S., Choi O., Horaud R.P. (2012). Time-of-Flight Cameras: Principles, Methods and Applications.

[10] Langmann B., Hartmann K., Loffeld O. Depth camera technology comparison and performance evaluation. Proceedings of the 1st International Conference on Pattern Recognition Applications and Methods ICPRAM 2012.

[11] Horaud R., Hansard M., Evangelidis G., Ménier C. (2016). An overview of depth cameras and range scanners based on time-of-flight technologies. Mach. Vis. Appl..

[12] Nejad S.M. (2006). Comparison of TOF, FMCW and Phase-Shift Laser Range-Finding Methods by Simulation and Measurement. Quart. J. Technol. Educ..

[13] Sackinger E. (2012). On the Noise Optimum of FET Broadband Transimpedance Amplifiers. IEEE Trans. Circuits Syst..

[14] Bielecki Z. (2002). Maximisation of signal-to-noise ratio in infrared radiation receivers. Opto-Electron Rev..

[15] Martin-Gorostiza E., Meca-Meca F.J., Lázaro-Galilea J.L., Salido-Monzú D., Martos-Nay E., Wieser A. Infrared local positioning system using phase differences. Proceedings of the 2014 Ubiquitous Positioning Indoor Navigation and Location Based Service (UPINLBS).

[16] Cho H.-S., Kim C.-H., Lee S.-G. (2014). A High-Sensitivity and Low-Walk Error LADAR Receiver for Military Application. IEEE Trans. Circuits Syst..

[17] Illade-Quinteiro J., Brea V.M., López P., Cabello D., Doménech-Asensi G. (2015). Distance Measurement Error in Time-of-Flight Sensors Due to Shot Noise. Sensors.

[18] Graeme J. (1995). Photodiode Amplifiers: OP AMP Solutions.

[19] Ruotsalainen T., Palojarvi P., Kostamovaara J. (2001). A wide dynamic range receiver channel for a pulsed time-of-flight laser radar. IEEE J. Solid-State Circuits.

[20] Instruments T. (2009). Transimpedance Considerations for High-Speed Amplifiers. https://e2e.ti.com/cfs-file/__key/telligent-evolution-components-attachments/00-14-00-00-00-00-02-68/sboa122-_2D00_-Transimpedance.pdf.

[21] Salido-Monzú D., Martín-Gorostiza E., Lázaro-Galilea J.L., Martos-Naya E., Wieser A. (2014). Delay Tracking of Spread-Spectrum Signals for Indoor Optical Ranging. Sensors.

[22] Noh J.-H. (2020). A Capacitive Feedback Transimpedance Amplifier with a DC Feedback Loop Using a Transistor for High DC Dynamic Range. Sensors.

[23] Hinaga S., Koledintseva M., Drewniak J.L., Koul A., Zhou F. Thermal Effects on PCB Laminate Material Dielectric Constant and Dissipation Factor. https://web.mst.edu/~marinak/files/my_publications/Papers/IPC_2010_S16_01_published_no_ppt.pdf.

[24] Salido-Monzú D., Meca-Meca F.J., Martín-Gorostiza E., Lázaro-Galilea J.L. (2016). SNR Degradation in Undersampled Phase Measurement Systems. Sensors.

[25] Baccar S., Lévi T., Dallet D., Shitikov V., Barbara F. A behavioral and temperature measurements-based modeling of an operational amplifier using VHDL-AMS. Proceedings of the 2010 17th IEEE International Conference on Electronics, Circuits and Systems.

